# Kindlin‐3 Promotes Angiogenesis via Notch Signalling and Is Crucial for Functional Recovery Postmyocardial Infarction

**DOI:** 10.1111/jcmm.70494

**Published:** 2025-03-18

**Authors:** Yan Sun, Wei Zheng, Xianling Liu, Kai Wang, Di Xu

**Affiliations:** ^1^ Department of Cardiology The First Affiliated Hospital with Nanjing Medical University Nanjing Jiangsu China; ^2^ Department of Geriatrics The First Affiliated Hospital with Nanjing Medical University Nanjing Jiangsu China

**Keywords:** angiogenesis, CMECs, Kindlin‐3, myocardial infraction, Notch

## Abstract

Angiogenesis is crucial for minimising ischemic injury postmyocardial infarction (MI), making it a significant target for cardioprotective therapies. While Kindlin‐3 has been linked to angiogenesis in breast cancer, its specific function in the context of MI remains largely unexplored. Although Kindlin‐3 has been implicated in breast cancer‐related angiogenesis, its role in MI remains underexplored. This study investigates the role of Kindlin‐3 in promoting angiogenesis, a process critical for cardiac recovery following MI. The study demonstrated a significant upregulation of Kindlin‐3 in cardiac microvascular endothelial cells (CMECs) in mice post‐MI. Overexpression of Kindlin‐3, achieved through cardiotropic adeno‐associated virus serotype 9 (AAV9) with the endothelial‐specific promoter Tie2, enhanced myocardial angiogenesis, improved cardiac function, decreased cardiomyocyte apoptosis and reduced fibrosis. In vitro, Kindlin‐3 overexpression promoted CMECs proliferation, migration, tube formation and the expression of angiogenesis‐related genes. Conversely, Kindlin‐3 knockdown exerted opposite effects. Mechanistically, Kindlin‐3 activated the Notch signalling pathway, as its effects were abrogated by the Notch inhibitor DAPT and β1 integrin knockdown. This study identifies Kindlin‐3 as a novel enhancer of angiogenesis and suggests its potential as a therapeutic target for myocardial repair.

## 1 Introduction

During myocardial infarction (MI), cardiomyocytes undergo irreversible death due to reduced blood flow caused by coronary ischemia [1]. This loss of cells is followed by the formation of fibrous scar tissue, which contributes to ventricular remodelling and ultimately leads to heart failure [2, 3]. Therapeutic angiogenesis has emerged as a promising strategy to restore cardiac function and mitigate the risk of heart failure post‐MI [4, 5]. Consequently, identifying key regulators of angiogenesis as potential therapeutic targets is crucial for improving blood flow recovery after MI.

Kindlins are a family of proteins that activate integrins and regulate cell adhesion, proliferation and migration [6, 7]. Kindlin‐1 is primarily expressed in epithelial cells and is involved in skin formation and repair [8]. Kindlin‐2, which is widely present in multiple cell types, such as endothelial cells [9] and fibroblasts [10], is critical for cell‐matrix adhesion, migration and proliferation [11]. Kindlin‐3 has been primarily investigated in haematopoietic cells, with its deficiency linked to immunodeficiency and abnormal platelet function [12], while it has also been identified as an endothelial protein in human umbilical vein endothelial cells, human dermal microvascular endothelial cells and human aortic endothelial cells [13]. However, Kindlin‐3 expression in CMECs and its role in postmyocardial infarction angiogenesis are not well understood.

The Notch signalling pathway, a key regulator of endothelial cell function [14], significantly impacts cardiovascular disease [15, 16] and enhances angiogenesis while mitigating ischaemia–reperfusion (I/R) injury during cardiac ischaemia [17, 18]. However, the influence of Kindlin‐3 on Notch signalling in endothelial cells has yet to be investigated.

Here, we investigate Kindlin‐3's role in promoting angiogenesis via the Notch signalling pathway and evaluate its importance for cardiac function recovery in a mouse model of MI. Through investigating the interaction between Kindlin‐3 and Notch, this research could provide new insights into therapeutic strategies for enhancing cardiac repair and recovery following ischaemic injury.

## 2 Materials and Methods

### 2.1 Myocardial Infarction (MI) Model, Adeno‐Associated Virus Infection and Experimental Groups

Male mice (4–5 weeks old) were used to eliminate the potential confounding effects of oestrogen‐dependent signalling, which are known to exert protective effects in myocardial ischemic injury, such as reducing post‐ischaemic cardiac dysfunction [19, 20]. The left anterior descending (LAD) coronary artery was ligated to induce MI, following established methods [21, 22, 23]. Mice were anaesthetised with isoflurane (1.5%–2%, RWD Life Science) inhalation and placed on artificial ventilation, after which the left anterior descending (LAD) artery was occluded, while sham surgeries followed the same procedures without LAD ligation. Cardiotropic adeno‐associated virus serotype 9 encoding Kindlin‐3 cDNA (AAV‐K3), driven by the endothelial cell‐specific promoter Tie2, was used to overexpress Kindlin‐3, with AAV‐NC serving as the control. Both AAV‐K3 and AAV‐NC were synthesised by Genomeditech. Adeno‐associated viruses at a dose of 1 × 10^12^ vg were injected into the tail veins 4 weeks prior to surgery. No mortality was observed due to LAD ligation or sham surgery. Four weeks after surgery, the mice were sacrificed (pentobarbital sodium 150 mg/kg, i.p.) for further analysis.

### 2.2 Echocardiography

Four weeks post‐LAD artery ligation or sham surgery, echocardiography, utilising the Vevo3100 system (VisualSonics, Toronto, Canada) equipped with a 35–40 MHz transducer, was used to assess cardiac function, following established protocols [24, 25]. Mice were anaesthetised with 1.5% isoflurane and positioned supinely on a heating pad with chest hair removed. Systolic and diastolic function parameters were derived from two‐dimensional M‐mode echocardiography and pulsed Doppler measurements. The individual who performed the echocardiographic analysis was blinded to the treatment.

### 2.3 Histology

Cardiac fibrosis was assessed by staining heart sections with Masson's trichrome reagent (Servicebio, G1006) following the manufacturer's instructions, quantifying fibrosis in the infarct border zone of the left ventricle as the ratio of collagen area to total tissue area [22]. To identify apoptotic cells in left ventricular samples, the Tunel Cell Apoptosis Detection Kit (Servicebio, G1504) was used following the manufacturer's instructions. Two independent, blinded investigators randomly selected six areas to count both TUNEL+ and total cells, calculating the apoptosis index as the ratio of TUNEL+ cells to total cells according to established protocols [22].

### 2.4 Immunohistochemistry (IHC)

After fixation and embedding, cardiac tissue sections were processed for indirect IHC staining and immunofluorescence (IF). IHC staining was performed using the HRP Kit (CWbio, CW2069S). Sections for IF staining were baked at 65°C for 2 h, deparaffinised with xylene, and rehydrated through a graded ethanol series, followed by rinsing in distilled water. Antigen retrieval was achieved using an enhanced citrate solution (Beyotime, P0083), after which sections were incubated with 3% hydrogen peroxide and blocked with Blocking Buffer (Beyotime, P0260). Primary antibodies were incubated with the sections overnight at 4°C, and secondary antibodies were applied at room temperature for 1 h. DAPI (Beyotime, C1005) was used to stain the nuclei.

### 2.5 CMEC Preparation

CMECs were extracted from myocardial tissues with the use of anti‐mouse CD31 microbeads, following previously described protocols [26]. The left ventricles underwent mincing followed by digestion with 0.1% collagenase type 2 (Worthington, LS004176) for three to five cycles. The resulting supernatant was collected and transferred to endothelial cell medium (ScienCell, 1001) to halt the enzymatic digestion. The solution was processed by passing it through a 100‐μm mesh filter, followed by collection and centrifugation at 1000 rpm for 8 min. ECM was used to resuspend the pellet. Following selection with anti‐mouse‐CD31 microbeads, CMECs were plated and grown for 2–3 days until they reached confluence and passaged further.

### 2.6 Cell Counting Kit‐8

Cell viability was assessed as previously described [27]. Briefly, CMECs were plated in 96‐well plates (6.0 × 10^3^ /well), followed by the addition of 10 μL of Cell Cycle Analysis Kit (Dojindo, CK04) to each well, which were then cultured in 90 μL of ECM; absorbance at 450 nm was recorded for each well after an additional 2–4 h.

### 2.7 Wound Healing Assay

The migration capacity of CMECs was assessed using a wound healing assay as previously described [28]. Briefly, monolayers of CMECs at 100% confluence were scratched using 200 μL pipette tips. The scratched areas were photographed immediately and 24 h after treatment. Wound closure percentage was quantified with ImageJ software.

### 2.8 Tube Formation Assay

To assess the angiogenic potential of CMECs, a tube formation assay was employed as previously described [29, 30]. Briefly, CMECs (1.5 × 10^4^/well) were seeded onto 96‐well plates coated with Matrigel (ABW, 0827904) and imaged at 40× magnification 6 h after seeding. The assessment of tube formation involved quantifying the total branch length and the number of master junctions per field using ImageJ software (version 1.8.0).

### 2.9 RT‐qPCR

Total RNA samples were isolated from left ventricles and CMEC cultures using TRIzol Reagent (Life Technologies, 15596‐018). This was followed by cDNA synthesis with a Vazyme reverse transcription kit (222‐01) and subsequent RT‐qPCR utilising ChamQ SYBR qPCR Master Mix (Vazyme, Q341‐02). All procedures were carried out according to the manufacturer's instructions, with GAPDH serving as the internal control. The PCR primers are provided in Table 1.

**TABLE 1:** List of genes used in this study.

Gene	Primer sequences
Kindlin‐3	F: 5′‐CTGACCTCTGAAGGAAGCAACT‐3′
	R: 5′‐CGGCTGTTTCTGCTAGCCTG‐3′
GAPDH	F: 5′‐AGGTCGGTGTGAACGGATTTG‐3′
	R: 5′‐TGTAGACCATGTAGTTGAGGTCA‐3′
Notch1	F: 5′‐GCTCCGAGGAGATCAACGAG‐3′
	R: 5′‐TTGACATCACCCTCACACCG‐3′
β1 integrin	F: 5′‐ATGCCAAATCTTGCGGAGAAT‐3′
	R: 5′‐ATGCCAAATCTTGCGGAGAAT‐3′

### 2.10 Western Blotting

Whole protein samples from the peri‐infarct area of the left ventricles or CMEC were extracted using RIPA buffer (Beyotime, P0013K) supplemented with a protease inhibitor cocktail (Bimake, B15001). The samples were then separated by SDS‐PAGE and transferred to PVDF membranes (Millipore, ISEQ00010). Following a 2–4‐h blocking of the membranes in 5% BSA (MP, 0218054950) at 4°C, overnight incubation with a primary antibody was conducted at the same temperature. This was succeeded by a 2‐h incubation with secondary antibodies. For protein detection, ECL reagent was utilised, and the resulting images of the target proteins were captured using a Tanon imaging system and analysed with ImageJ (version 1.8.0). Details of the antibodies used can be found in Table 2.

**TABLE 2:** List of antibodies used in this study.

Name of antibody	Manufacturer	Identifier	Concentration
Kindlin‐3	Affinity Biosciences	DF13558	1:1000 (WB), 1:200 (IHC)
VEGF	Proteintech	19003‐1‐AP	1:1000 (WB)
β1‐integrin	Bioss	bs‐0486R	1:2000 (WB), 1:400 (IHC)
GAPDH	Proteintech	6004‐1‐Ig	1:2000 (WB)
Hif‐1α	Affinity Biosciences	BF8002	1:2000 (WB)
eNOS	Proteintech	27120‐1‐AP	1:1000 (WB)
p‐eNOS	Proteintech	28937‐1‐AP	1:1000 (WB)
CD31	Proteintech	11265‐1‐AP	1:1000 (WB), 1:400 (IF)
Cleaved Caspase 3	Cell Signaling Technology	9664	1:1000 (WB)
Caspase 3	Cell Signaling Technology	9662	1:1000 (WB)
α‐Actinin	Sigma	A7811	1:400 (IF)
α‐SMA	Proteintech	14395‐1‐AP	1:400 (IF)
vWF	Proteintech	66682‐1‐Ig	1:400 (IF)
Notch1	Proteintech	20687‐1‐AP	1:1000 (WB), 1:400 (IHC)
Hes1	Affinity Biosciences	DF7569	1:1000 (WB)
HRP‐conjugated a Goat Anti‐Rabbit	Servicebio	GB233303	1:200 (IHC)
Goat Anti‐Rabbit IgG, Peroxidase Conjugated, H + L	Biosharp	BL003A	1:10000 (WB)
Goat Anti‐Mouse IgG, Peroxidase Conjugated, H + L	Biosharp	BL001A	1:10000 (WB)
Alexa Fluor 488‐conjugated AffiniPure Goat Anti‐Rabbit IgG (H + L)	Jackson	115‐585‐003	1:200 (IF)
Alexa Fluor 594‐conjugated AffiniPure Goat Anti‐Mouse IgG (H + L)	Jackson	115‐585‐003	1:200 (IF)

Abbreviations: IF, immunofluorescence; IHC, immunohistochemistry; WB, Western blot.

### 2.11 Immunofluorescence Cell Staining

CMECs, cultured in 24‐well plates to reach 40%–50% confluence, were first fixed for 15 min with 4% paraformaldehyde and then permeabilized using 0.2% Triton X‐100 for 15 min. After permeabilization, the cells were blocked with 5% goat serum albumin for 1 h at room temperature, followed by overnight incubation at 4°C with primary antibodies. Fluorescence‐conjugated secondary antibodies were applied to the cells, followed by DAPI staining the next day.

### 2.12 Statistical Analysis

Statistical analyses were conducted using GraphPad Prism software 8.0, with data represented as mean ± SEM. The analyses included one‐way or two‐way ANOVA and unpaired Student's *t*‐test, with a *p*‐value of less than 0.05 deemed statistically significant.

## 3 Results

### 3.1 Kindlin‐3 Expression in CMECs From MI Mouse Hearts

**FIGURE 1:**
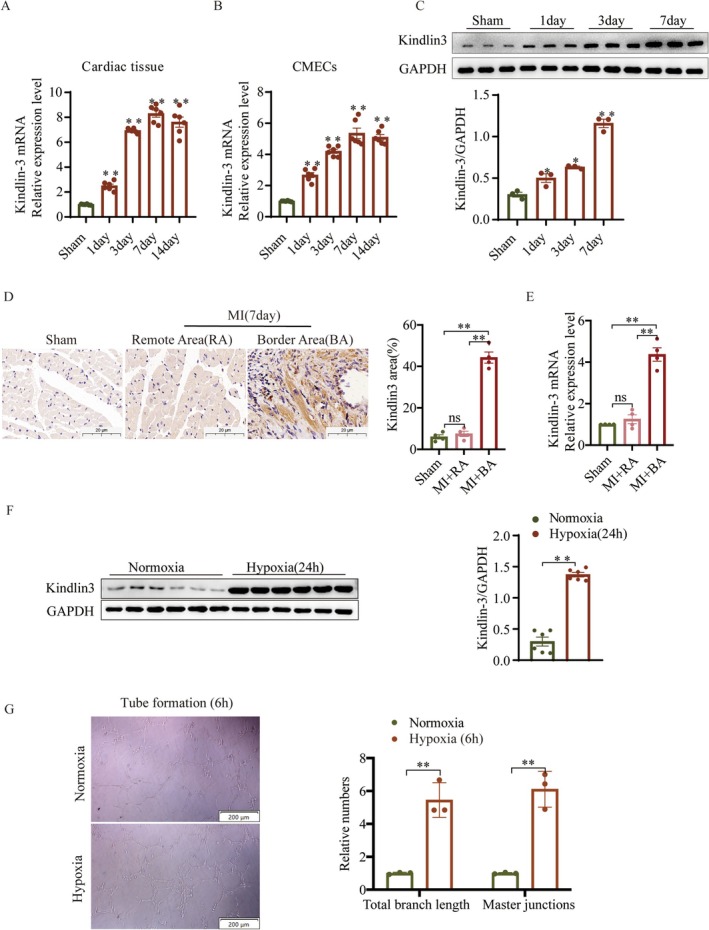
Kindlin‐3 expression increases in CMECs after MI. (A, B) Quantitative PCR (qPCR) analysis showing Kindlin‐3 mRNA expression in cardiac tissues and CMECs from the infarct border zone at different time points post‐MI (*n* = 6). (C) Representative images and quantitative analysis of Kindlin‐3 protein expression in CMECs from the infarct border area post‐MI (*n* = 3). (D) Representative immunohistochemical staining of Kindlin‐3 in Sham‐operated and myocardial infarction (MI) mice, including the remote area (RA) and border area (BA) post‐MI (Scale bars = 20 μm). Kindlin‐3 expression levels were quantified and compared (*n* = 4). (E) qPCR analysis of Kindlin‐3 mRNA expression in Sham‐operated and MI mice, including the remote area (RA) and border area (BA) post‐MI (*n* = 4). (F) Immunoblotting analysis showing representative images and quantitative data of Kindlin‐3 expression in CMECs under hypoxia or normoxia conditions (*n* = 6). (G) The effects of hypoxia on tube formation were assessed using a Matrigel assay (*n* = 3). Data were presented as mean ± SEM. *p < 0.05, **p < 0.01 versus sham or normoxia group.

We examined Kindlin‐3 expression in myocardial tissue and CMECs post‐MI to understand its role. The findings indicated a notable rise in Kindlin‐3 expression, reaching a peak on day 7 post‐MI (Figure 1A). To validate this finding, CMECs were isolated from the heart tissues of both sham and MI mice and characterised via immunofluorescence (Figure S1A). qPCR and Western blot analyses further demonstrated that CMECs from the infarct border zone showed significantly elevated Kindlin‐3 levels compared to the sham group, reaching its maximum on day 7 post‐MI (Figure 1B,C). Immunohistochemistry (IHC) demonstrated that Kindlin‐3 expression was markedly higher in the border area (BA) post‐MI compared to the remote area (RA) and sham groups (Figure 1D). Similarly, qPCR analysis revealed a significant upregulation of Kindlin‐3 expression in the BA post‐MI compared to both the RA and sham groups (Figure 1E). To investigate the function of Kindlin‐3 further, in vitro experiments were conducted on CMECs under hypoxic conditions to induce tube formation, while Kindlin‐3 expression was measured. Results showed that hypoxia increased both tube formation and Kindlin‐3 expression in CMECs compared to normoxia (Figure 1F,G). These findings suggest that elevated Kindlin‐3 levels in CMECs may contribute to the pathophysiology of MI and have potential clinical relevance.

### 3.2 Overexpression of Kindlin‐3 Enhanced Cardiac Function and Mitigated Pathological Remodelling Post‐MI

**FIGURE 2:**
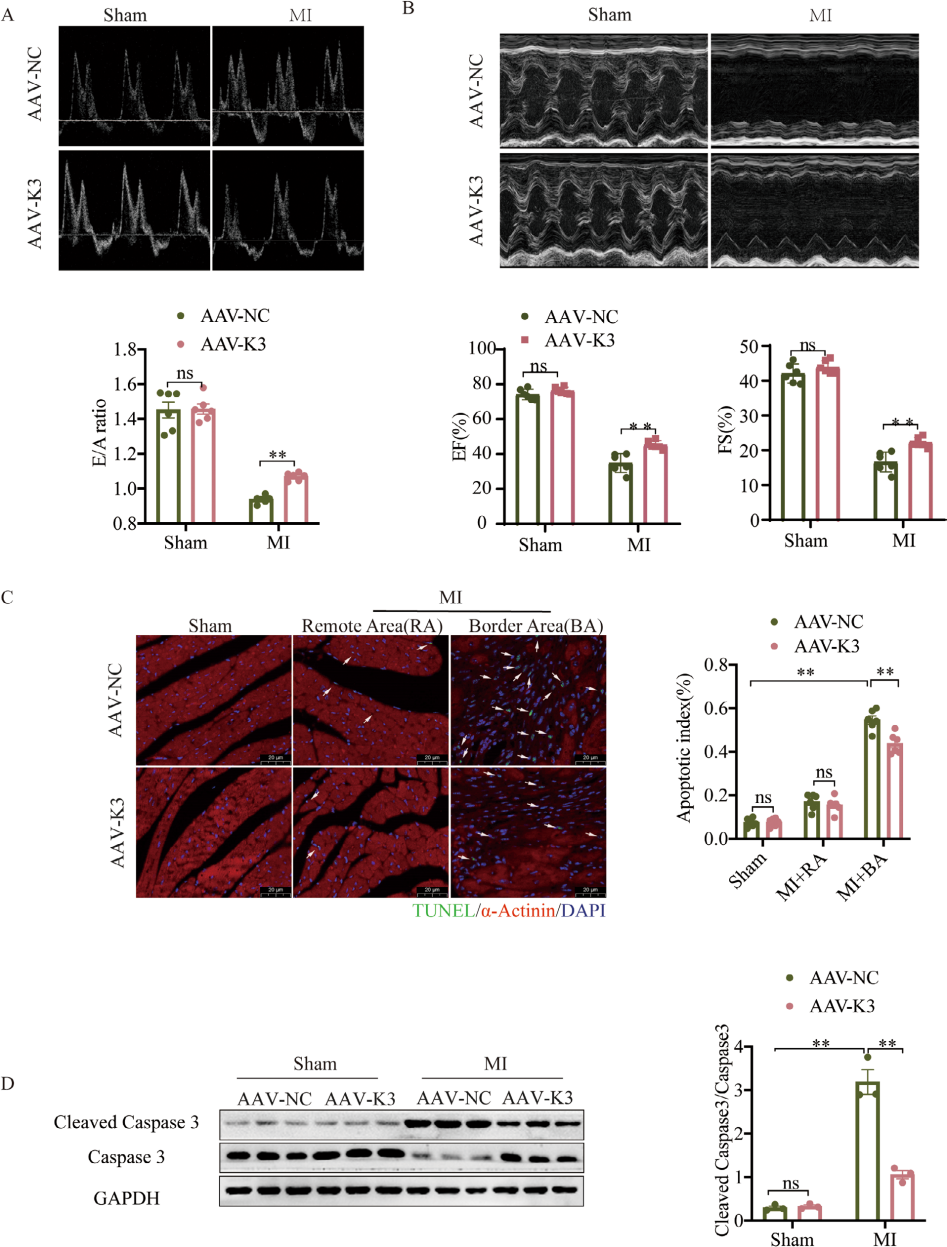
Kindlin‐3 treatment improves cardiac function and reduces cardiomyocyte apoptosis in the post‐MI border zone. Kindlin‐3 was administered via the tail vein as described in the Materials and Methods section. (A) The peak early filling to late diastolic ventricular filling velocity (E/A ratio) was used to assess cardiac diastolic function, (B) while ejection fraction (EF%) and fractional shortening (FS%) were used to evaluate cardiac systolic function (*n* = 6). (C) Representative images of TUNEL and nuclear (DAPI) staining in cardiomyocytes (α‐actinin) from AAV‐NC and AAV‐K3 mice post‐MI or Sham operation, including the remote area (RA) and border area (BA). Arrows indicate TUNEL‐positive (green) cardiomyocyte (red) nuclei (blue; Scale bars = 20 μm; *n* = 6). (D) Representative Western blot images and quantitative analysis showing the ratio of Cleaved Caspase 3 to Caspase 3 in mice 28 days post‐MI (*n* = 3). ns, not significant. Data were presented as mean ± SEM. **p < 0.01.

**FIGURE 3:**
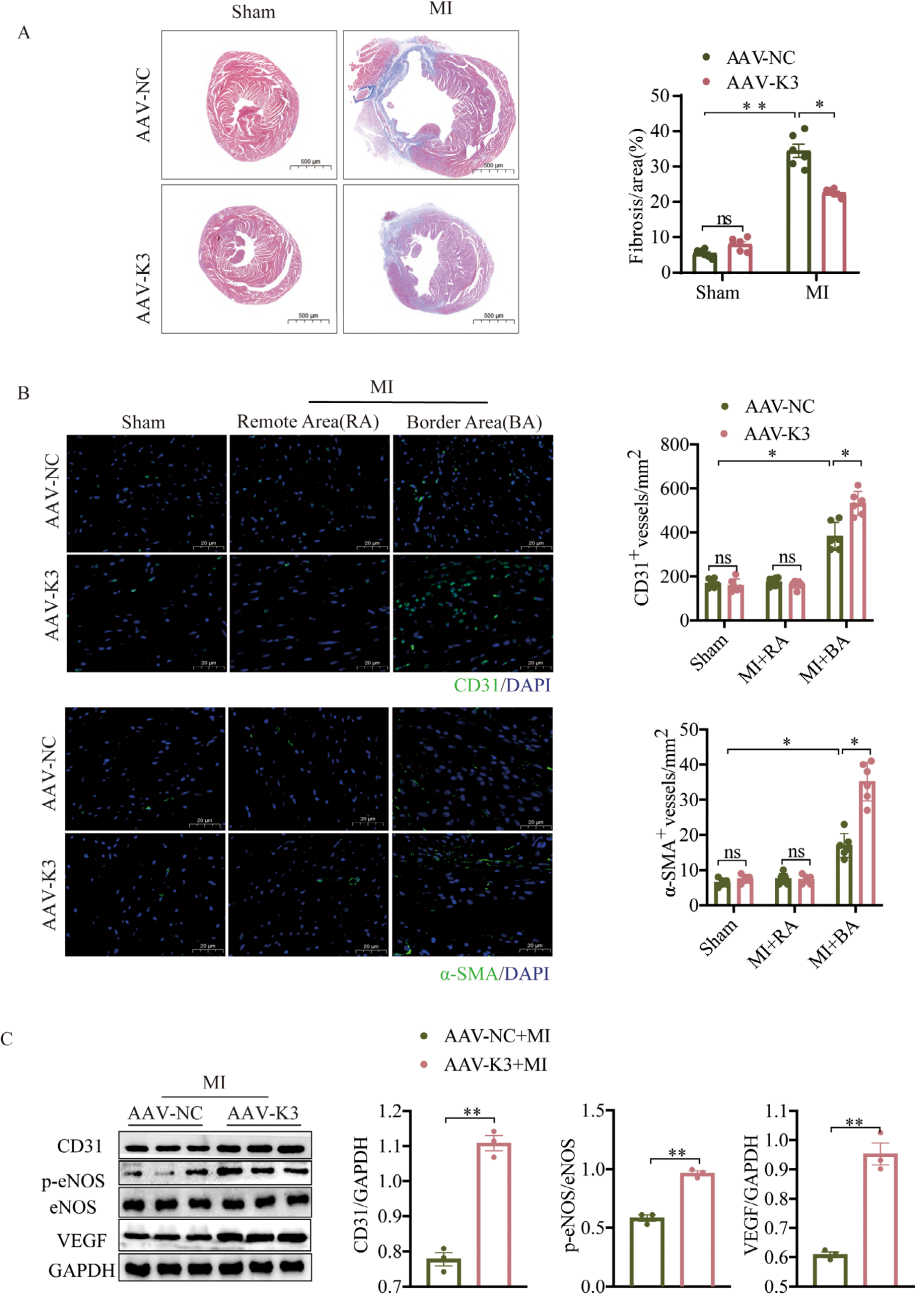
Kindlin‐3 promotes angiogenesis in vivo. (A) Representative images of Masson's trichrome staining and quantitative data illustrating the extent of fibrosis in heart sections from the indicated groups (*n* = 6). Scale bars = 500 μm. (B) Four weeks post‐MI, ventricular tissues were analysed using immunofluorescence staining to evaluate capillary (CD31) and arteriolar density (α‐SMA) in AAV‐NC and AAV‐K3 mice post‐MI or Sham operation, including the remote area (RA) and border area (BA, *n* = 6). (C) Representative images and quantitative analysis of Western blots demonstrate the expression of angiogenesis‐related proteins in mice 28 days post‐MI (*n* = 3). Data were presented as mean ± SEM. ns, not significant, *p < 0.05, **p＜0.01.

To assess whether Kindlin‐3 contributes to angiogenesis and functional recovery post‐MI, we constructed an AAV9 vector encoding Kindlin‐3 expression, regulated by the Tie2 promote (AAV‐K3) (Figure S2A). A concentration of 1 × 10^12^ vg/mL was selected and infected into mice 4 weeks before MI via tail vein (Figure S2B). Subsequent analyses showed that AAV‐K3 significantly increased Kindlin‐3 expression in CMECs, with no detectable expression in cardiomyocytes (CMs) (Figure S2C). Echocardiography was performed on mice to evaluate cardiac function. At 28 days post‐MI, AAV‐K3‐treated mice showed improved diastolic and systolic function compared to the AAV‐NC group, as evidenced by increased E/A ratio, EF% and FS% (Figure 2A,B). Additionally, adverse cardiac remodelling caused by MI was alleviated in AAV‐K3‐treated mice, reflected by reduced LVIDd and LVIDs (Figure S3A). As shown in Figure 2C, the number of TUNEL‐positive cardiomyocytes in the border area post‐MI was significantly reduced in AAV‐K3 mice compared to AAV‐NC mice. Consistently, the protein levels of the pro‐apoptotic molecule cleaved caspase‐3 were also lower in the left ventricle of AAV‐K3 mice compared to AAV‐NC mice following MI (Figure 2D). Furthermore, Masson's trichrome staining demonstrated a marked reduction in fibrosis in the AAV‐K3 + MI group compared to the AAV‐NC + MI group (Figure 3A). These findings support the hypothesis that Kindlin‐3 overexpression not only enhances left ventricular (LV) function but also mitigates progressive LV remodelling.

### 3.3 Kindlin‐3 Enhanced Angiogenesis In Vivo and In Vitro

Angiogenesis plays a crucial role in reducing cell death and fibrosis in ischemic hearts [31]. To assess the impact of Kindlin‐3 on post‐MI angiogenesis, we examined capillary (CD31) and arteriole (α‐SMA) density in MI heart tissue. Immunostaining revealed significantly higher capillary and arteriole density in the border area of AAV‐K3 + MI hearts compared to AAV‐NC + MI hearts 4 weeks post‐MI (Figure 3B). Western blot analysis further confirmed increased levels of angiogenesis‐related proteins, including CD31, VEGF and phosphorylated eNOS (p‐eNOS), in the AAV‐K3 + MI group (Figure 3C). These findings suggest that Kindlin‐3 overexpression promotes angiogenesis after MI.

**FIGURE 4:**
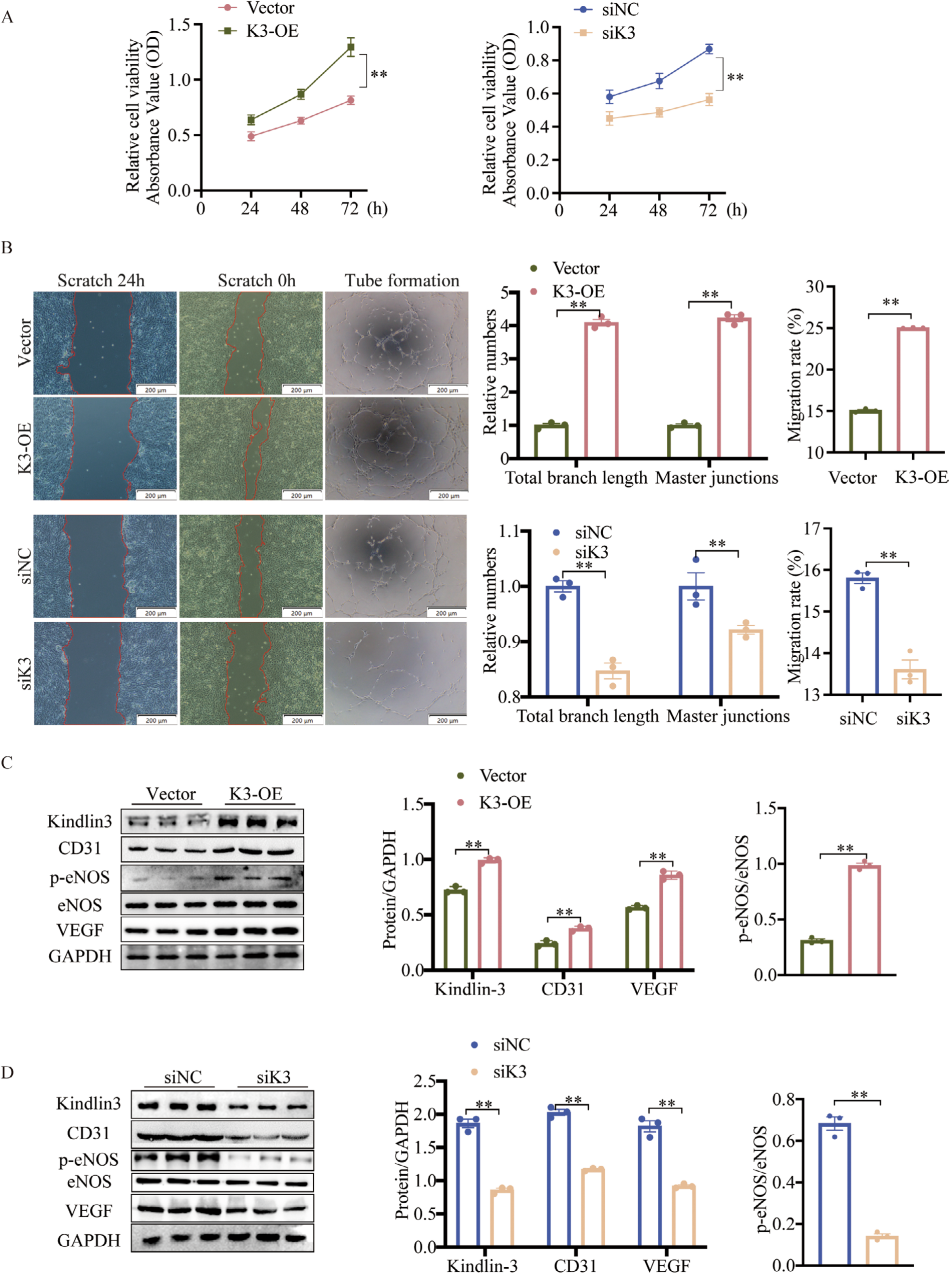
Kindlin‐3 promotes angiogenesis in vitro. (A) Cell viability in CMECs was analysed using the CCK‐8 assay at 0, 24 and 72 h following Kindlin‐3 overexpression or knockdown (*n* = 3). (B) Scratch assays and tube formation were assessed through representative images and quantitative analysis under conditions of Kindlin‐3 overexpression or knockdown (*n* = 3). (C) Representative Western blot images and quantitative analysis demonstrating Kindlin‐3 protein levels and angiogenesis‐related protein expression in CMECs following transfection with Kindlin‐3 overexpression plasmids (*n* = 3). (D) Representative Western blot images and quantitative analysis showing Kindlin‐3 protein levels and angiogenesis‐related protein expression in CMECs after transfection with siRNA targeting Kindlin‐3 (*n* = 3). Data were presented as mean ± SEM. **p < 0.01.

The upregulation of Kindlin‐3 in CMECs during tube formation suggests its potential role in angiogenesis. Kindlin‐3 expression was modified using overexpression plasmids or siRNA, with transfection efficiency confirmed via Western blot analysis (Figure S4A). Since viability, migration and tube formation are key processes in angiogenesis [32], we performed a series of functional assays. The CCK‐8 assays revealed a significantly increased CMEC viability in the Kindlin‐3 overexpression (K3‐OE) group compared to the vector group after 72 h (Figure 4A). Consistent results were observed in cellular morphological studies (Figure S4B). Additionally, the wound healing assay revealed enhanced CMECs migratory ability in the K3‐OE group (Figure 4B), while matrigel assays confirmed that Kindlin‐3 overexpression significantly enhanced tube formation. Conversely, Kindlin‐3 knockdown impaired these angiogenic processes (Figure 4B). Given the strong regulation of angiogenesis by angiogenic genes [33], we analysed the expression of VEGF, CD31, p‐eNOS and eNOS in CMECs. Following 24 h of Kindlin‐3 overexpression, VEGF, CD31 and p‐eNOS protein levels were upregulated (Figure 4C). In contrast, Kindlin‐3 knockdown reduced the expression of these proteins (Figure 4D). Overall, these findings indicate that Kindlin‐3 promotes angiogenesis in CMECs and may contribute to vascular regeneration following MI.

### 3.4 Kindlin‐3 Promotes Angiogenesis Through Notch Signalling Activation

**FIGURE 5:**
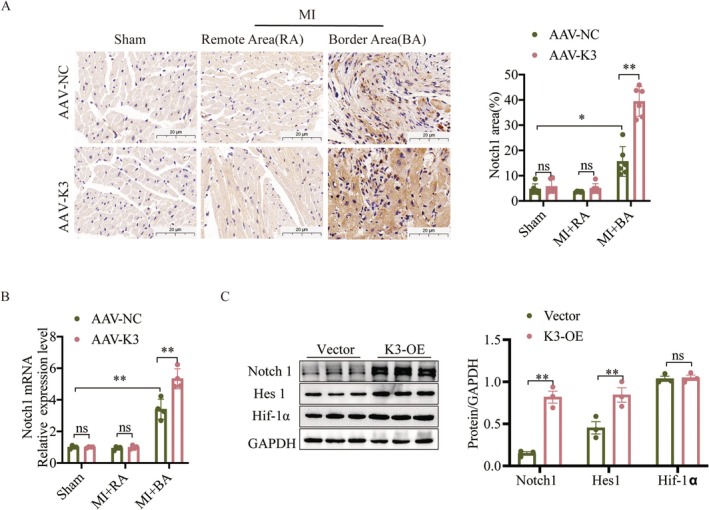
Kindlin‐3 overexpression activates the Notch signalling pathway. (A) Representative immunohistochemical staining of Notch1 in AAV‐NC and AAV‐K3 mice post‐MI or Sham operation, including the remote area (RA) and border area (BA). (Scale bars = 20 μm). Notch1 expression levels were quantified and compared (*n* = 6). (B) qPCR analysis of Notch1 mRNA expression in AAV‐NC and AAV‐K3 mice post‐MI or Sham operation, including the remote area (RA) and border area (BA) (*n* = 4). (C) Representative Western blot images and quantitative analysis showing Notch1, Hes1, and Hif‐1α protein levels in CMECs. ns, not significant. Data were presented as mean ± SEM. **p < 0.01, *p < 0.05.

**FIGURE 6:**
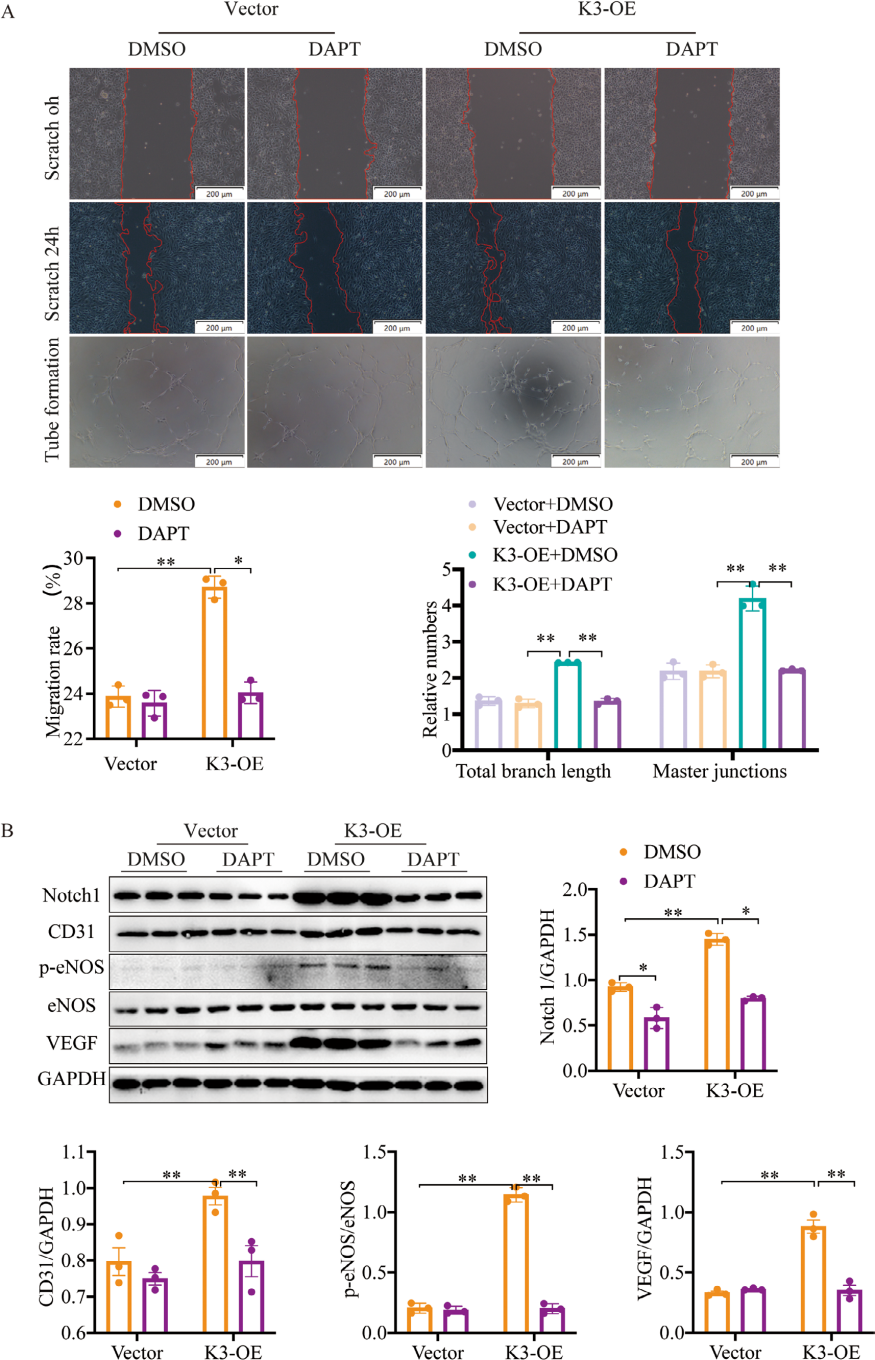
Inhibition of the Notch pathway abolishes Kindlin‐3 enhanced angiogenesis. (A) DAPT treatment's impact on migration and tube formation was examined in Vector and K3‐OE CMECs (*n* = 3). (B) Representative images and quantitative analysis of Western blots demonstrate Notch1 and angiogenesis‐related protein levels in CMECs. DAPT 10 μM. Data were presented as mean ± SEM. *p < 0.05, **p < 0.01.

Transcription factors such as Hif‐1α and Notch regulate angiogenic gene secretion [34, 35]. As shown in Figure 5A,B, MI led to an upregulation of Notch1 expression in the border area, which was significantly enhanced by AAV‐K3. Subsequent analyses revealed that overexpression of Kindlin‐3 in CMECs increased the expression of Notch1 and Hes1, but not Hif‐1α, compared to the vector group (Figure 5C). To assess whether Notch signalling mediates Kindlin‐3‐induced angiogenesis, we treated CMECs with 10 μM DAPT, a Notch pathway inhibitor, for 2 h following a 24‐h transfection with Kindlin‐3 overexpression plasmids. CCK‐8 assays and morphological analyses showed that DAPT treatment significantly suppressed Kindlin‐3‐induced viability and proliferation of CMECs (Figure S5A,B). Additionally, the increased migratory capacity observed in Kindlin‐3 overexpression CMECs was inhibited by DAPT treatment (Figure 6A). Similarly, DAPT reduced the tube‐forming capacity of CMECs stimulated by Kindlin‐3 overexpression (Figure 6A). Western blot analysis confirmed that DAPT reversed the Kindlin‐3‐induced upregulation of Notch1, VEGF, CD31 and p‐eNOS (Figure 6B). These findings imply that Kindlin‐3 facilitates angiogenesis via activation of the Notch signalling pathway.

### 3.5 Kindlin‐3 Regulates Notch Signaling Is β1‐Integrin Dependent

**FIGURE 7:**
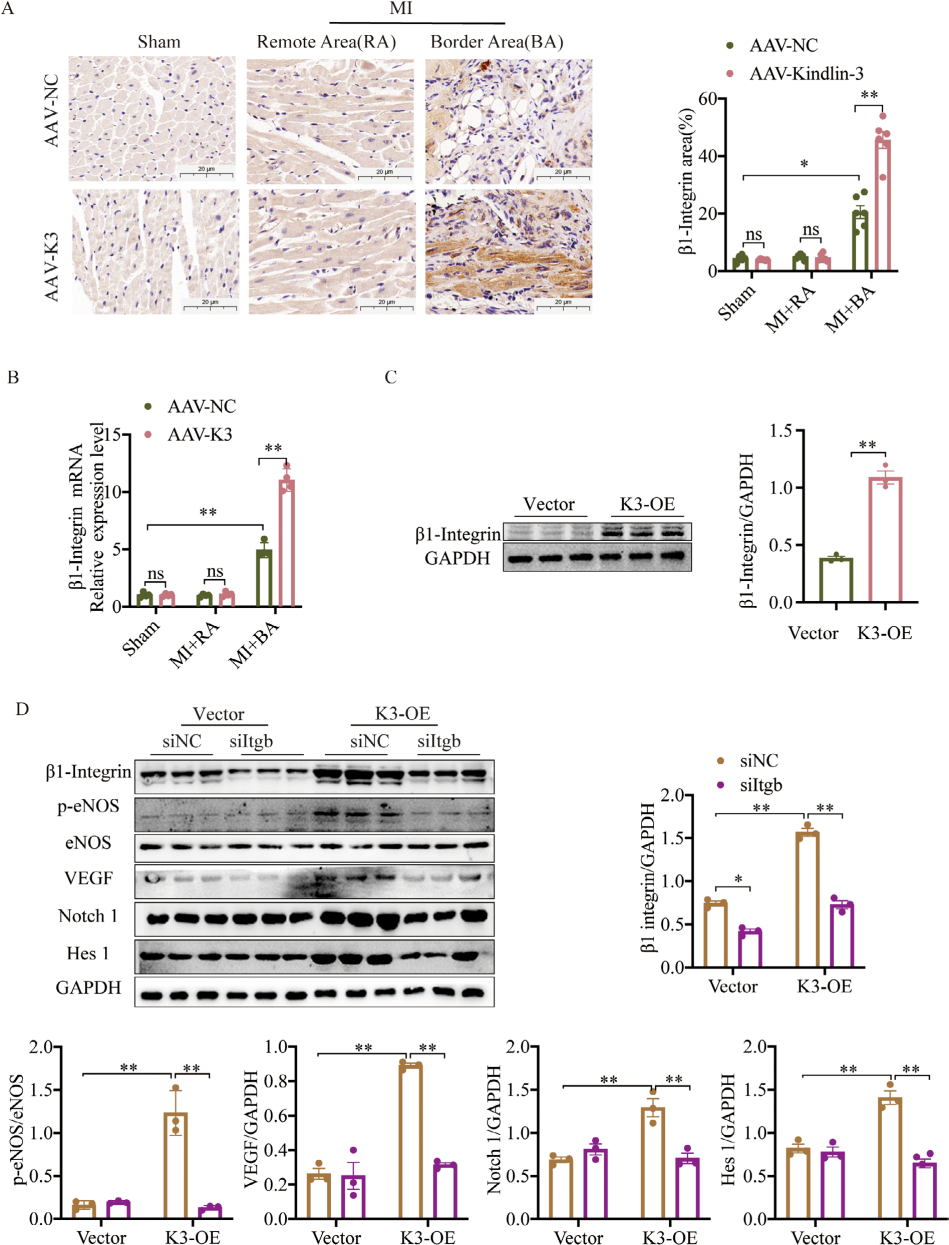
Kindlin‐3 modulates Notch signalling through a β1 integrin‐dependent mechanism in vitro. (A) Representative immunohistochemical staining of β1 Integrin in AAV‐NC and AAV‐K3 mice post‐MI or Sham operation, including the remote area (RA) and border area (BA). (Scale bars = 20 μm). β1 Integrin expression levels were quantified and compared (*n* = 6). (B) qPCR analysis of β1 Integrin mRNA expression in AAV‐NC and AAV‐K3 mice post‐MI or Sham operation, including the remote area (RA) and border area (BA). (*n* = 4). (C) Quantitative analysis and representative Western blot images of β1‐Integrin in CMECs following treatment with Vector or K3‐OE (*n* = 3). (D) The expression of the specified proteins was evaluated through Western blotting after a 2‐h treatment with the Notch inhibitor DAPT. (*n* = 3). ns, not significant. Data were presented as mean ± SEM. **p < 0.01, *p < 0.05.

To further investigate the mechanism by which Kindlin‐3 influences angiogenesis, we examined the role of its potential receptor. β1‐Integrin expression was increased in the border area after MI and was further elevated by AAV‐K3 treatment (Figure 7A,B). Similarly, Kindlin‐3 overexpression in CMECs enhanced β1‐Integrin levels (Figure 7C). To determine whether β1‐Integrin is required for Kindlin‐3‐mediated Notch signalling activation, we used β1‐Integrin siRNA (siItgb) to suppress its expression in CMECs. Knockdown of β1‐Integrin abolished the Kindlin‐3‐induced upregulation of notch1, Hes1, VEGF and p‐eNOS in CMECs, which was eliminated following β1‐Integrin knockdown (Figure 7D). These findings indicate that Kindlin‐3 promotes Notch signalling in a β1‐Integrin‐dependent manner.

## 4 Discussion

This study shows that Kindlin‐3 is upregulated during myocardial infarction (MI), promotes angiogenesis, reduces cardiomyocyte apoptosis and mitigates cardiac fibrosis, which are crucial for post‐MI recovery. We examined Kindlin‐3's role in angiogenesis in vitro to understand the underlying mechanisms. Kindlin‐3 expression increases during endothelial tubule formation. Modulating Kindlin‐3 levels in CMECs demonstrated that overexpression enhances cell proliferation, migration and tube formation, whereas downregulation suppresses these activities. Kindlin‐3‐induced angiogenesis was linked to the stimulation of the β1 integrin‐dependent Notch signalling pathway.

Kindlin‐3 is a vital component of the Kindlin family that facilitates integrin‐mediated cell adhesion, migration and signalling [6]. Emerging evidence highlights Kindlins as potential therapeutic targets for angiogenic diseases [9, 36]. For example, embryos lacking endothelial Kindlin‐2 die from haemorrhage due to defective angiogenesis [9]， while kindlin‐3 has been linked to tumour angiogenesis via the Twist pathway [37]. Kindlin‐3 is also strongly associated with cardiovascular diseases. Bjoern F and colleagues conducted a study showing that Kindlin‐3 undergoes cleavage in human platelets during ST‐elevation myocardial infarction, highlighting its potential as a platelet marker in vascular ischaemia [38]. Additionally, increased Kindlin‐3 expression has been observed in human atherosclerotic plaques, particularly in M2 macrophages [39]. Similarly, Yu et al. identified Kindlin‐3 expression in plaques and highlighted its association with plaque erosion, distinguishing it from plaque rupture in STEMI cases [40]. While previous research has predominantly focused on Kindlin‐3 expression in haematopoietic cells, it has also been detected in non‐haematopoietic cells, including endothelial cells [13]. This study identified increased levels of Kindlin‐3 in CMECs from mouse hearts post‐MI, which were associated with enhanced recovery of cardiac function. This suggests that Kindlin‐3‐dependent pathways may promote angiogenesis and aid in heart repair following myocardial infarction.

Angiogenesis refers to the development of new blood vessels and can occur in physiological processes, such as wound healing, as well as in pathological conditions, including tumour growth [41]. In myocardial infarction, it helps restore blood flow and provides oxygen and nutrients, highlighting its significance in modern medicine [42, 43]. VEGF members are key regulators of angiogenesis. VEGF‐A primarily drives angiogenesis via VEGFR‐2, whereas VEGF‐B, through VEGFR‐1, helps prevent cardiomyocyte apoptosis and supports cardiac function after MI [44]. Current challenges include enhancing wound healing post‐MI by targeting angiogenesis. Our study shows that Kindlin‐3 is highly expressed in CMECs isolated from MI mouse hearts, thereby promoting angiogenesis. In vitro, the overexpression of Kindlin‐3 promotes endothelial cell proliferation, migration, tube formation and the expression of genes linked to angiogenesis, establishing Kindlin‐3 as a novel angiogenesis activator, although the specific regulatory mechanisms are still not fully understood.

Research shows that Notch1 signalling in the adult heart is inactive under normal conditions but becomes transiently active after MI or other stressors [45]. Prior studies indicated that Kindlin‐2 in endothelial cells promotes angiogenesis through Notch signalling [9]. Our findings reveal that Kindlin‐3 overexpression in CMECs upregulates Notch1 and Hes1. Blocking Notch signalling negates Kindlin‐3's effects on cell proliferation, migration and the expression of genes linked to angiogenesis, underscoring Notch signalling's crucial role in Kindlin‐3‐mediated angiogenesis.

β1‐Integrin, a transmembrane protein, is essential for regulating endothelial cell proliferation, migration and signalling, which are all vital for angiogenesis [46, 47]. Kindlin‐3, an activator of integrins, interacts with DDR1 to modulate β1‐integrin activation [48]. Our study demonstrates that the lack of β1‐integrin negates the impact of Kindlin‐3 on endothelial cell function and angiogenesis.

This study has several limitations. Previous research has highlighted the role of phosphorylated Kindlin‐3 in integrin activation and tumour angiogenesis [49]. Future studies should elucidate whether Kindlin‐3 promotes post‐MI angiogenesis through phosphorylation and identify the specific phosphorylation sites involved. Additionally, since immune cells influence cardiac remodelling [50], further studies should explore whether Kindlin‐3 enhances angiogenesis and reduces remodelling by modulating immune function.

## 5 Conclusion

This study is the first to demonstrate that Kindlin‐3 promotes angiogenesis after myocardial infarction, a key process for tissue repair in mice. We identified its role and mechanism in enhancing angiogenesis and highlighted its potential as a therapeutic target for ischaemic vascular diseases.

## Author Contributions

**Yan Sun:** data curation (lead), formal analysis (lead), software (lead), validation (lead), writing – original draft (lead), writing – review and editing (lead). **Wei Zheng:** data curation (lead), methodology (lead), supervision (lead), writing – original draft (lead). **Xianling Liu:** data curation (equal), resources (equal), supervision (equal), visualization (equal). **Kai Wang:** conceptualization (lead), data curation (lead), methodology (equal), writing – review and editing (lead). **Di Xu:** conceptualization (lead), funding acquisition (lead), project administration (lead), writing – review and editing (lead).

## Ethics Statement

For this study, C57BL/6 mice were sourced from the Nanjing Medical University Animal Center and kept in the institution's Animal Laboratory Resource Facility. All animal procedures and the isolation of cardiac cells from myocardial tissue were approved by the Institutional Animal Care and Use Committee at Nanjing Medical University (Approval NO. IACUC‐2311032) and the procedures adhered to the National Institutes of Health guidelines for the Care and Use of Laboratory Animals.

## Consent

The authors have nothing to report.

## Conflicts of Interest

The authors declare no conflicts of interest.

## Supporting information


**Figure S1.** Representative immunofluorescence images showing vWF and CD31 expression in cultured mouse CMECs.
**Figure S2.** AAV9‐mediated Kindlin‐3 overexpression in vivo.
**Figure S3.** Echocardiographic assessment of cardiac structure post‐MI.
**Figure S4.** Kindlin‐3 expression and its effects on CMEC density after transfection.

## Data Availability

The authors will provide the raw data that underlie the conclusions of this article upon request, without any limitations.
