# Supplementary figures and images for: Kindlin‐3 Promotes Angiogenesis via Notch Signalling and Is Crucial for Functional Recovery Postmyocardial Infarction
Source: J Cell Mol Med. 2025 Mar 18;29(6):e70494. doi: 10.1111/jcmm.70494 (PMC11915616; doi:10.1111/jcmm.70494)

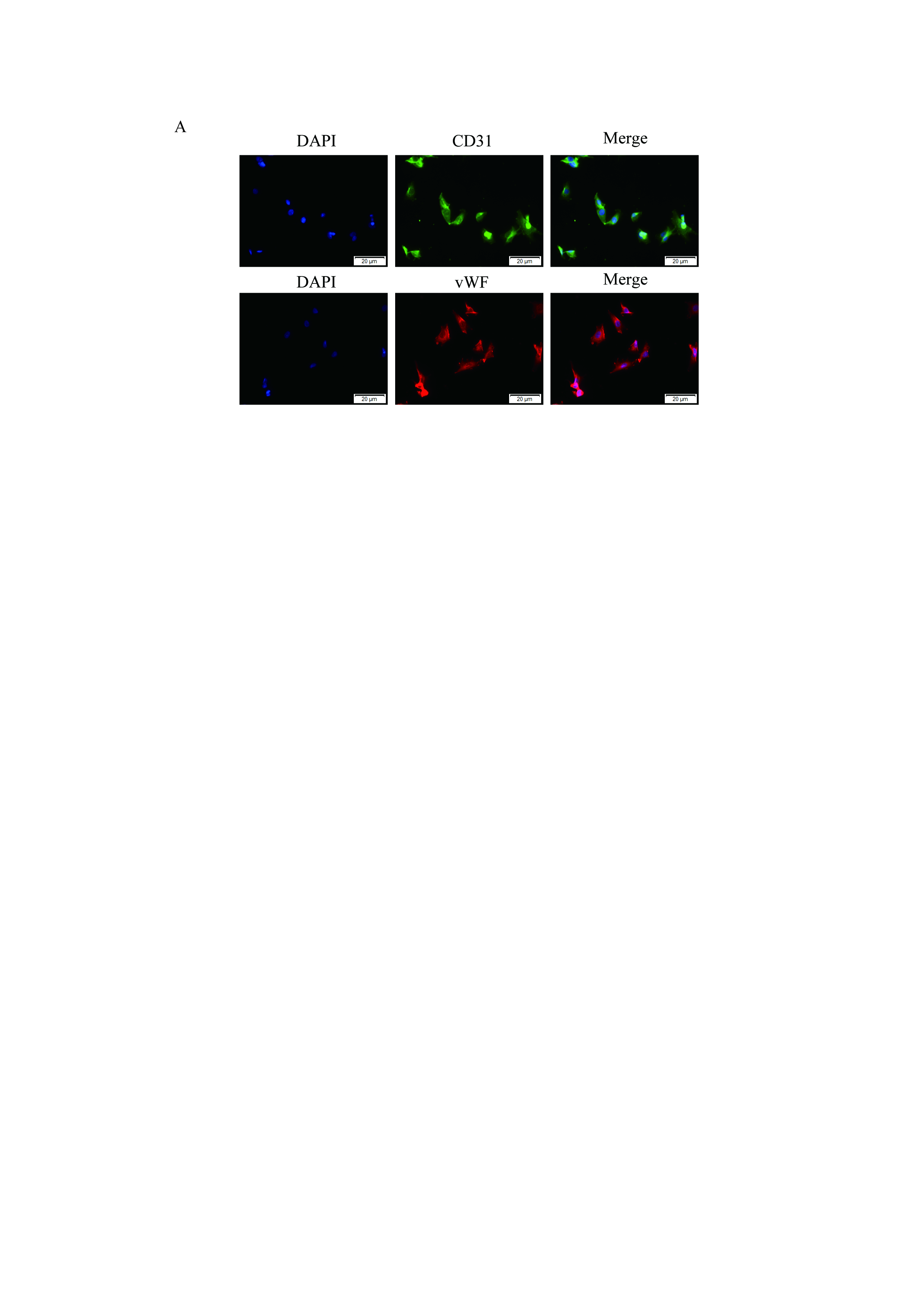

Supplement: Supplementary file 1 — Figure S1. Representative immunofluorescence images showing vWF and CD31 expression in cultured mouse CMECs. Figure S2. AAV9‐mediated Kindlin‐3 overexpression in vivo. Figure S3. Echocardiographic assessment of cardiac structure post‐MI. Figure S4. Kindlin‐3 expression and its effects on CMEC density after transfection. [file JCMM-29-e70494-s001.zip › FigureS1-S4/Suppl.1.tif]

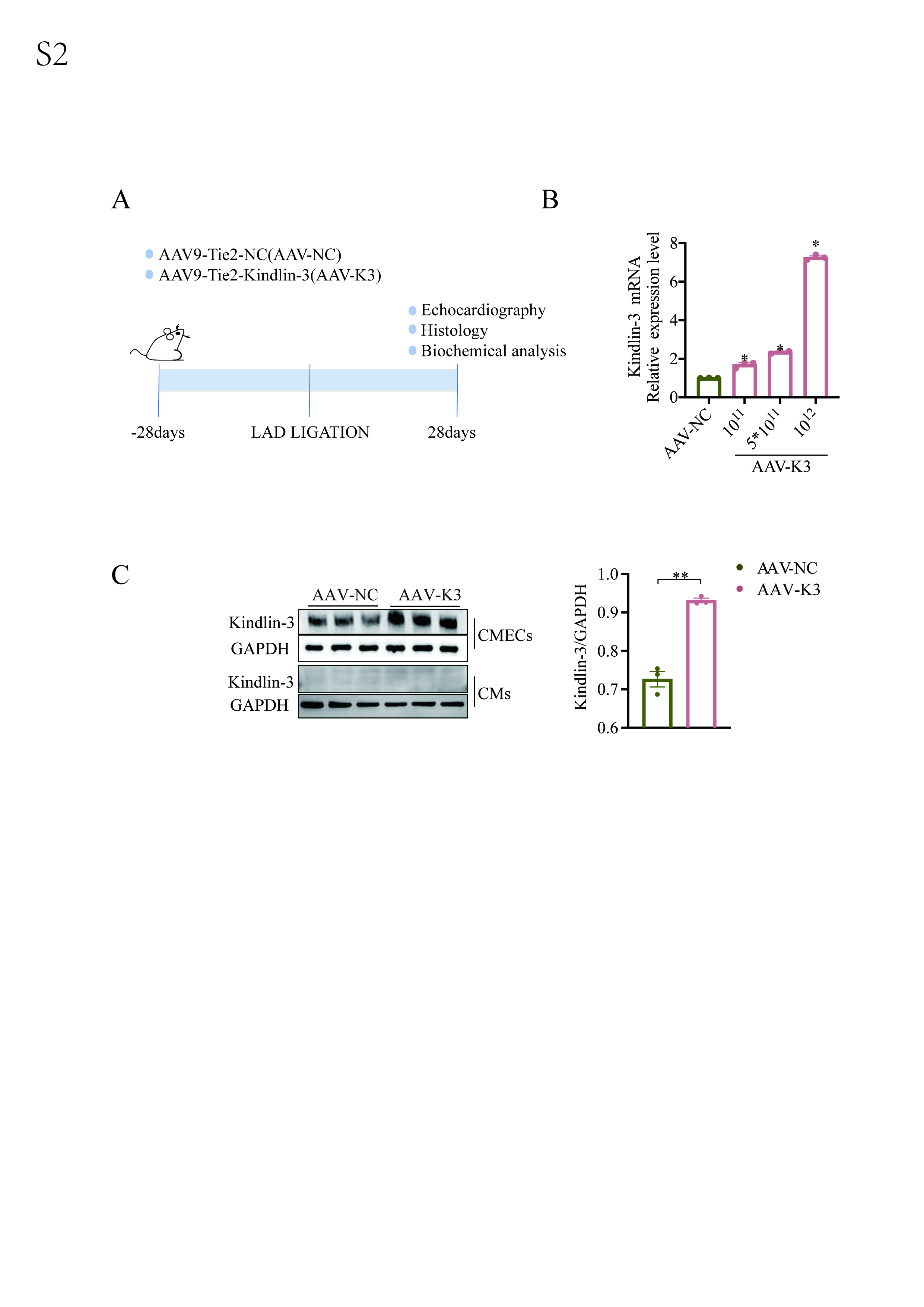

Supplement: Supplementary file 1 — Figure S1. Representative immunofluorescence images showing vWF and CD31 expression in cultured mouse CMECs. Figure S2. AAV9‐mediated Kindlin‐3 overexpression in vivo. Figure S3. Echocardiographic assessment of cardiac structure post‐MI. Figure S4. Kindlin‐3 expression and its effects on CMEC density after transfection. [file JCMM-29-e70494-s001.zip › FigureS1-S4/Suppl.2.tif]

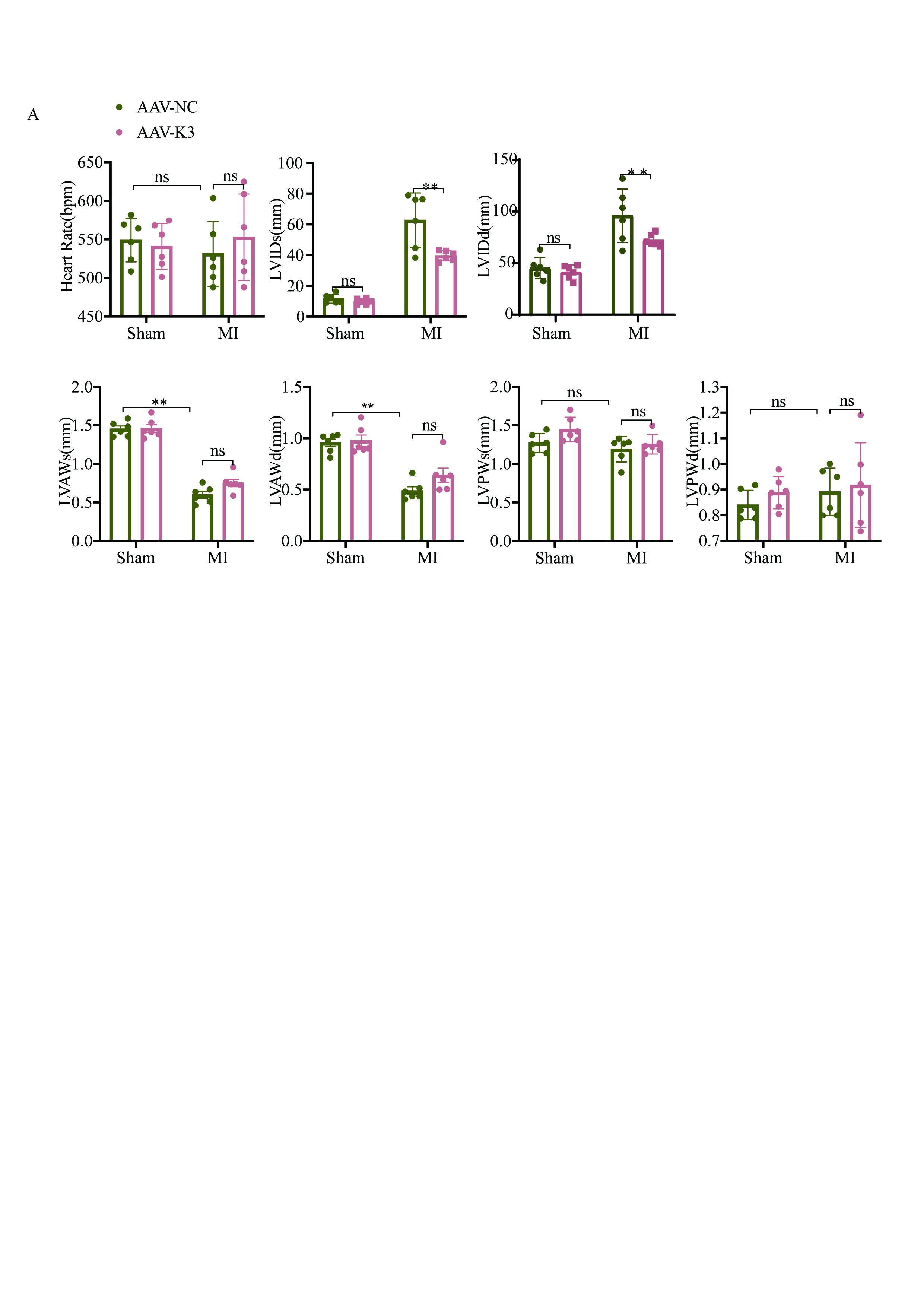

Supplement: Supplementary file 1 — Figure S1. Representative immunofluorescence images showing vWF and CD31 expression in cultured mouse CMECs. Figure S2. AAV9‐mediated Kindlin‐3 overexpression in vivo. Figure S3. Echocardiographic assessment of cardiac structure post‐MI. Figure S4. Kindlin‐3 expression and its effects on CMEC density after transfection. [file JCMM-29-e70494-s001.zip › FigureS1-S4/Suppl.3.tif]

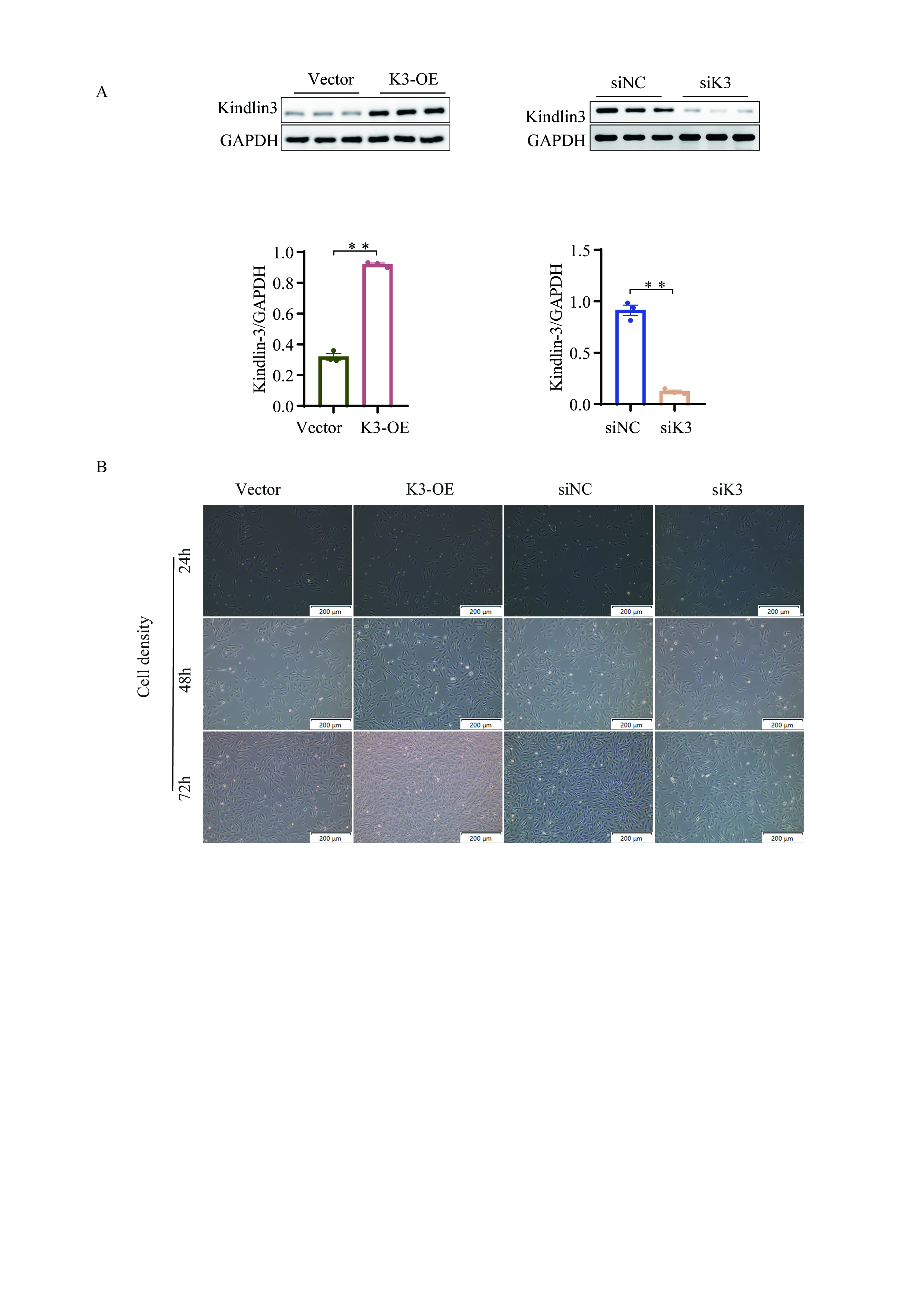

Supplement: Supplementary file 1 — Figure S1. Representative immunofluorescence images showing vWF and CD31 expression in cultured mouse CMECs. Figure S2. AAV9‐mediated Kindlin‐3 overexpression in vivo. Figure S3. Echocardiographic assessment of cardiac structure post‐MI. Figure S4. Kindlin‐3 expression and its effects on CMEC density after transfection. [file JCMM-29-e70494-s001.zip › FigureS1-S4/Suppl.4.tif]
